# Guiding of laser pulses in plasma waveguides created by linearly-polarized femtosecond laser pulses

**DOI:** 10.1038/s41598-018-21392-z

**Published:** 2018-02-16

**Authors:** N. Lemos, L. Cardoso, J. Geada, G. Figueira, F. Albert, J. M. Dias

**Affiliations:** 1 Lawrence Livermore National Laboratory, NIF and Photon Sciences, 7000 East Avenue, Livermore, California 94550 USA; 2 GoLP/Instituto de Plasmas e Fusão Nuclear, Instituto Superior Técnico, Universidade de Lisboa, 1049-001 Lisbon, Portugal

## Abstract

We experimentally demonstrate that plasma waveguides produced with ultra-short laser pulses (sub-picosecond) in gas jets are capable of guiding high intensity laser pulses. This scheme has the unique ability of guiding a high-intensity laser pulse in a plasma waveguide created by the same laser system in the very simple and stable experimental setup. A hot plasma column was created by a femtosecond class laser that expands into an on-axis parabolic low density profile suitable to act as a waveguide for high intensity laser beams. We have successfully guided ~10^15^ W cm^−2^ laser pulses in a 8 mm long hydrogen plasma waveguide with a 35% guiding efficiency.

## Introduction

Many applications for high-intensity laser-plasma interaction benefit from a large interaction length, in which the laser beam maintains its intensity over a few centimeters. These applications include laser-driven wakefield accelerators^1,2^, X-ray lasers^3,4^ and harmonic generation^5^. The laser plasma interaction length of a focused beam is mainly limited by diffraction and by ionization induced refraction^6^ on the order of the Rayleigh length (for non-relativistc laser intensities). In the last 20 years, several schemes for guided laser propagation have been demonstrated to overcome these limitations. One of them is the plasma waveguide. The modified refractive index profile of the plasma balances the diffractive spreading and refraction, thus the intense laser pulse is guided at a constant radius over many Rayleigh ranges. Critical parameters for most of the applications are the length of the waveguide, capacity to control the guided mode, the life time of the waveguide, reproducibility of the waveguide and good coupling and transmission. Therefore it is very challenging for guiding schemes to meet all these requirements.

The guiding methods developed to date can be divided into three classes: hollow capillary structures, self-focused propagation and preformed plasma waveguides. The first method has been used in mono-mode^7,8^ and multimode guiding^9,10^ where the best results showed a transmission of 30% with a 1.2 *cm* capillary and 5% with a 4 *cm* capillary both filled with helium and guiding laser intensities up to 10^15^ *W*/*cm*^2^. In such waveguides, high-quality injected modes and exceptionally accurate alignment is needed or a misalignment at the entrance will result in efficiency loss and eventually damaging the capillary. Self-focused propagation^11–15^ relies on relativistic self-focusing, where relativistic enhancement of electron inertia near beam center lowers the on-axis plasma density (refraction index is increased) and ponderomotive charge displacement by the laser pulse. These effects require optical intensities higher than 10^18^ W cm^−2^ and the propagation lengths achieved are smaller that 1.5 cm with a maximum transmission of 70%^15^. The maximum propagation length is strongly limited by nonlinear effects such as the the erosion of the leading edge of the laser pulse^11^. Preformed density plasmas can act as waveguides when the electron density increases with radius from the beam propagation axis. Thus the index of refraction is peaked on axis, slowing the phase front and continuously balancing diffraction. Preformed plasma waveguides have been achieved by ionizing and heating a linear region of gas, either by optical ionization or by high-voltage discharge ionization^16–20^. The optical ionization schemes use long laser pulses (~10–100 ps) with linear focusing optics in gas jets^21–23^, or short laser pulses focused in gas jets with clusters^24^ or without clusters^25^. The initial plasma heating generates a hydrodynamic expansion wave leading to density depletion in the center of the plasma column forming the plasma waveguide. Guided propagation of high-intensity pulses was first demonstrated in preformed channels generated by focusing a long laser pulse (tens of picoseconds) with an axicon in a gas jet^21^. Optical guiding was observed over 24 Rayleigh lengths with 77% energy transmition. Latter this technique was shown to guide laser pulses with intensities of 5 × 10^16^ W cm^−2^ over 9 Rayleigh lengths with 52% energy transmission^26^. With the high-voltage discharges inside capillaries method, Butler *et al*.^17^ report guiding of laser pulses with peak input intensities greater than 10^17^ W cm^−2^ in 30 mm and 50 mm long H-filled capillaries. It was observed optical guiding of 36% (82% of the initial energy beam energy) and 23% (70% of the initial energy beam energy) of the input intensity, corresponding to a guiding of 42 Rayleigh lengths in the 5 cm case. When using short pulses to create plasma waveguides Kumarappan *et al*.^24^ reported the production of centimeter long channels in an argon cluster jet, where a subsequent pulse was guided with 3 × 10^17^ W cm^−2^ intensity and 50% energy guiding efficiency over 40 Rayleigh lengths. Recent results^25^ show that it is possible to generate plasma waveguides in a hydrogen and helium gas jet with no clusters using a femtosecond class laser. We showed that ultra-short intense laser pulses (sub-picosecond) can heat the plasma through the Above Threshold Ionization (ATI) effect, allowing the generation of plasma waveguides. In quasi-classical ATI at sub-relativistic laser intensities the tunnel ionized electrons gain a drift velocity that is related to the phase of the laser electric field at the moment it is ionized. This results in an energy spread which increases the plasma temperature. In previous experimental studies^25^, we have shown that as the plasma column expands, a parabolic density profile is generated on-axis. This demonstrates that ultra-short pulses can indeed heat the plasma to high enough initial temperatures to create plasma waveguides suitable for guiding high intensity laser beams.

In this work we present a study of the guiding properties of waveguides generated by short pulses in a hydrogen gas jet. To create the plasma waveguides an experiment has been conducted by focusing a ~400 fs laser pulse into a 8 mm hydrogen gas jet. A temporal characterization of the expanding plasma column has been carried out to show that the plasma column expands into an on-axis parabolic density profile suitable as a waveguide for high intensity laser beams. A second pulse is then injected into the plasma waveguide with a controllable delay and we characterized the injected laser pulse at the exit of the plasma waveguide. This paper is organized as follows. In Sec. II the experimental setup is described. The experimental results, including characterization of the plasma waveguides and the guided beam is discussed in Sec. III, before concluding in Sec. IV.

## Results

### Experimental setup

This experiment has been performed at the L2I laboratory at IST. A laser beam was produced by a chirped pulse amplification laser system with energy of 70 *mJ* (on target), duration of 400 *fs*, central wavelength of 1053 *nm*, a laser shot every 90 s and an initial beam diameter of 2 cm.

**Figure 1:**
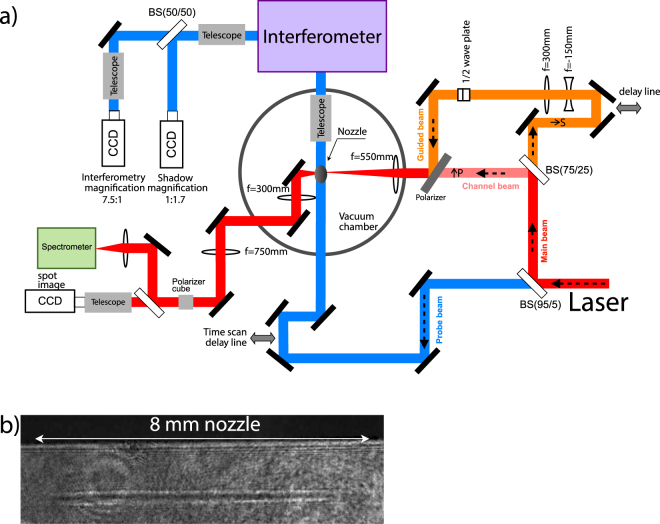
(**a**) Experimental setup used showing an example a shadowgraph at time zero (**b**).

Figure 1(a) shows a schematic of the experimental arrangement and the optical diagnostics used in the experiment. The laser was divided in two beams: the main beam containing 95% of the beam energy and the probe beam containing 5% of the energy. The main beam is further divided into the channel beam containing 75% of the energy of the main beam and the guided beam containing the remaining 25% of the energy. The channel beam is focused with a f/28 lens after the gas jet in order compensate the ionization induced refraction and create a plasma column that will expand and create a plasma waveguide. The guided beam passes through a beam expander that increases the beam diameter to 4 cm to reduce the spot size to match the plasma waveguide guiding mode. Then, the guided beam passes through a half-wave plate rotating the polarization by 90°, so that it can later be distinguished from the channel beam. The beam is then focused collinearly with the channel beam by the same lens with NA = f/14 to the entrance of the gas jet. The focus position of the guided beam is controlled by slightly changing the distance between the two expanding lenses. The delay between the two beams is controlled by a delay line in the path of the guided beam. The probe beam is used to probe the plasma transversely in an interferometer, which can generate both interferometry, to measure the plasma electron density profile, and shadowgraphy, to determine the plasma shape and length. The temporal evolution of the plasma density from 0 ns to 3 ns is studied by varying the probe beam delay.

The spots of the channel and guided beams are imaged onto a digital CCD camera (14 bit), with a magnification of 7.5:1 and a resolution of 0.6 *μ*m/pixel, by two telescopes. To distinguish the two beams, a cube polarizer is used between the telescopes to select the beams imaged onto the CCD camera. To measure the transmitted spectral changes, a portion of the guided beam is transmitted into an optical spectrometer. The CCD cameras and the spectrometer are externally triggered with an electronic shutter that integrates the image for a few tens of ms. The hydrogen gas jet is produced by an 8 mm Mach 6 Laval nozzle^27^.

### Generation of the plasma waveguide

Using the experimental setup shown in Fig. 1(a) it has been possible to create and fully characterize the guiding capabilities of the plasma waveguides in a hydrogen plasma. To create a long and uniform plasma column we took advantage of the ionization induced refraction effect^6^ to maintain the beam diameter throughout the length of the nozzle. Therefore, we focused the beam at the end of the nozzle to compensate for ionization induced refraction.

**Figure 2:**
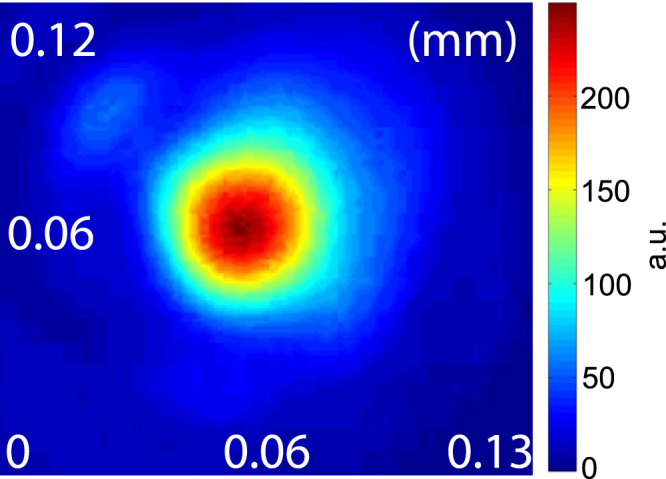
Channel beam intensity profile at the entrance of the gas jet.

As an example, Fig. 1(b) shows a shadowgraph image when the channel beam just leaves the nozzle (delay zero), where a uniform 8 mm plasma column is created. Figure 2 shows the channel beam intensity profile at the entrance of the nozzle with a spot size of 50 *μ*m (at the 1/*e*^2^ intensity point) corresponding to an intensity of 5 × 10^15^ W cm^−2^.

**Figure 3:**
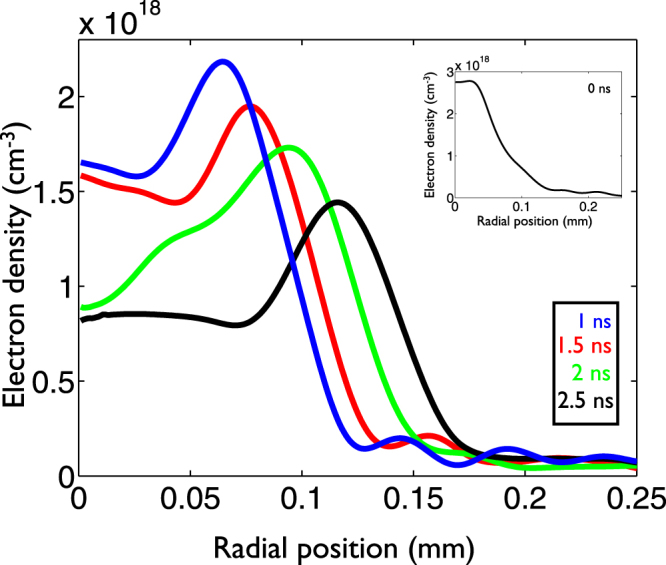
Temporal evolution of the electron plasma density averaged over the longitudinal direction for a hydrogen plasma column created in an 8 mm nozzle with a backup pressure of 20 bar. The inset shows the initial plasma density profile.

The temporal evolution of the created plasma column is measured by varying the delay line of the probe beam (Fig. 1). Using an interferogram analysis code it is possible to determine the plasma density profiles for each probe beam delay line position. The interferogram analysis code consists of two steps: First it retrieves the phase shift of the interferograms, and then calculates the refraction index from the phase shift. To obtain the phase shift we have applied an automatic method based on two-dimensional FFTs. The final result of the phase shift caused by the gas jet is obtained by subtracting the phase maps of the reference interferogram (without plasma) from the phase given by the interferogram with plasma in order to eliminate the fringe bending caused by the optical components of the experimental setup. The extraction of the electron density profiles was done inverting the 2D phase maps using an Abel inversion algorithm. The plasma densities averaged over 3 shots and over the longitudinal direction for several delays with a backing pressure of 20 bar are shown in Fig. 3. Although the interferometry diagnostic only analyses the plasma density on a 1 mm window around the center of the nozzle the shadowgraphy diagnostic confirms that the plasma channel extends for 8 mm (Fig. 1(b)).

The peak electron density shown in the inset of Fig. 3 was created by a laser pulse with an intensity of 5 × 10^15^ W cm^−2^ (Fig. 2) well above the necessary 5 × 10^14^ W cm^−2^ to fully ionize the hydrogen molecule. Since recombination for low density plasmas with temperatures of tens of eV and low ion charges occurs on a time scale of tens or hundreds of nanoseconds^28,29^, the ionization level is maintained for the full range of delays measured here.

When the laser leaves the gas, a hot column of plasma is left behind (inset of Fig. 3). The heated electrons pull the cold ions radially outwards and the plasma expands at the local ion sound speed^25,30^. The ions in the expanding front collide with both the ions and atoms in the relatively cold and weakly ionized gas (plasma-gas interface). These collisions lead to a density build-up at the plasma-gas interface forming a shock wave that leaves a density depression at the center of the plasma. A shock wave is formed when a disturbance propagates at a speed higher that the local sound speed. In our scenario the plasma expansion velocity is much higher than the sound speed in the surrounding gas leading to the formation of a shock wave^29^. After a few hundred picoseconds the plasma begins to expand and by 1 ns there is a clear shock structure formed at the edges of the plasma (Fig. 3). At the center of the plasma, an on-axis parabolic low density profile is formed that is suitable to act as a waveguide for high intensity laser beams.

### Guiding properties of the plasma waveguide

For each time delay of the waveguide expansion it is possible to calculate the matched spot size (W_0_)^13^ supported by the waveguide. Considering that the density profile at the center of the plasma waveguide can be approximated by a parabola given by n_*e*_ = n_0_ + *α* r^2^, where n_0_ is the maximum plasma density (at the shock wave position), r is the radial position and $\alpha =\tfrac{{\rm{\Delta }}n}{{W}_{0}^{2}}$, it is possible to calculate the matched spot size.

For the delays presented on Fig. 3 the matched spot sizes are the following: 20, 22, 27 and 34 *μ*m for 1, 1.5, 2 and 2.5 ns respectively. To test the guiding ability of these waveguides a second pulse is injected into the waveguide and a complete characterization of the guided laser pulse at the exit is carried out. The guided beam is focused into the entrance of the waveguide to match the guiding mode. The focus position is optimized by changing the distance between the two expanding lenses shown in Fig. 1(a). Also it is possible to control the time delay between the channel pulse and the guided beam with a delay line (Fig. 1(a)) to select the best plasma waveguide profile to match the spot of the guided beam.

**Figure 4:**
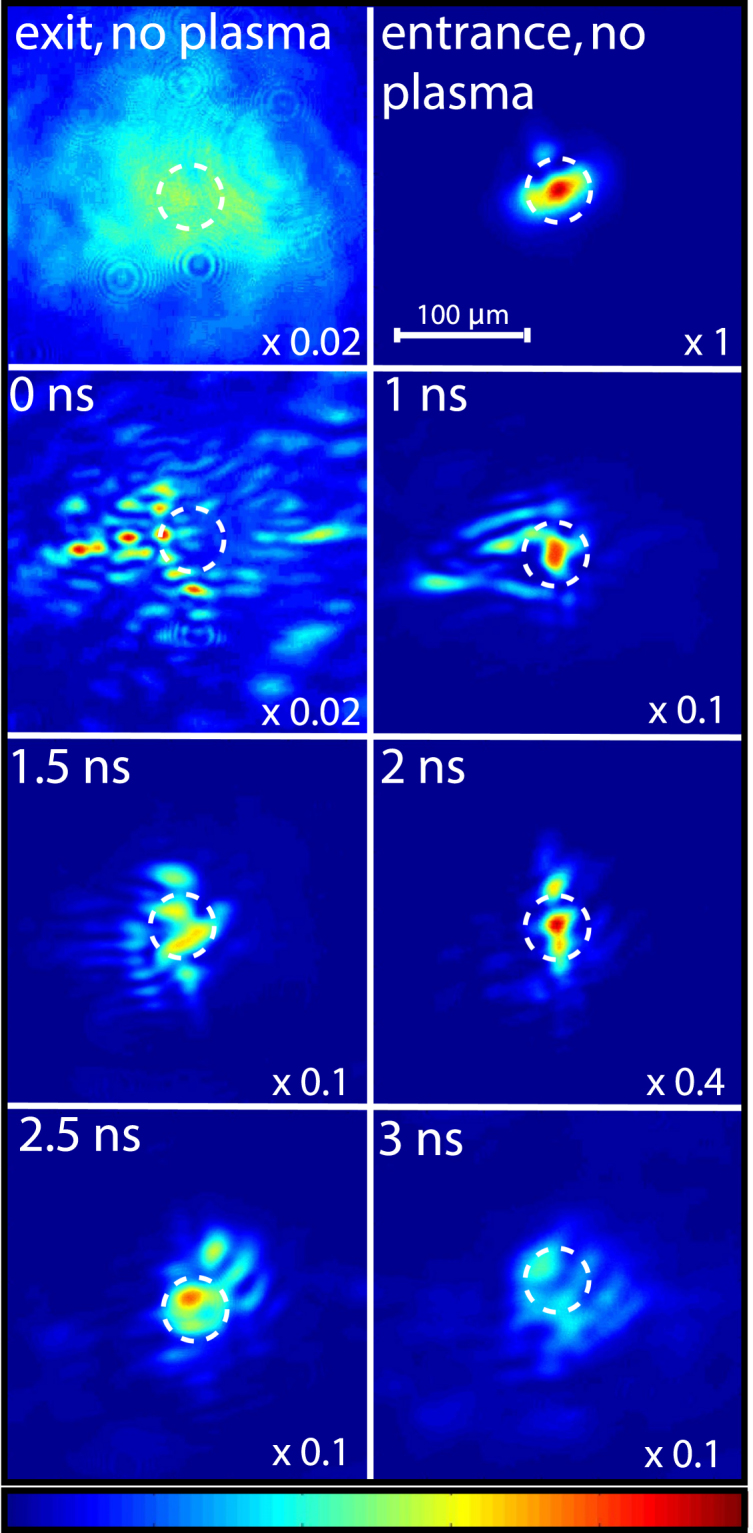
Top two images images represent the intensity profile of the guided beam at the exit and entrance (left and right) of the plasma waveguide when there is no initial plasma (no channel beam). The white circle represents the spot size of 25 *μ*m of the entrance guided beam. The other images show the guided beam intensity profile at the waveguide exit, when there is an initial plasma formed, for several delays. The color table of all images is adjusted by a scale factor indicated at the bottom right corner of each image.

**Figure 5:**
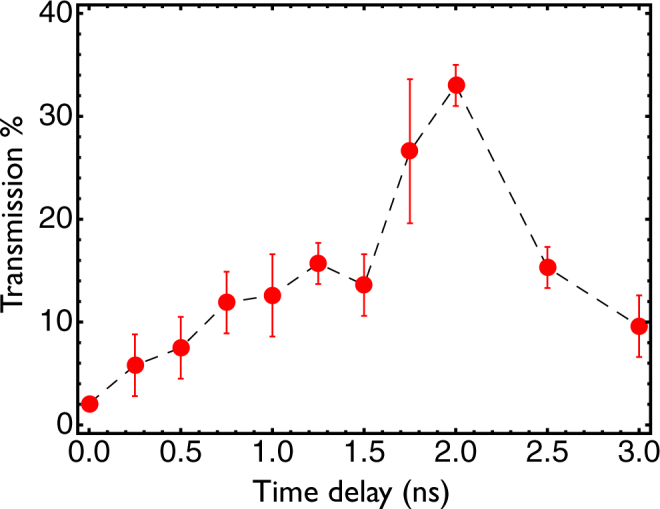
Percentage of the encircled energy of the guided beam transmitted through the plasma waveguide in a radius of 25 *μ*m for several delays between the channel and guided beams. The light transmission is normalized to the amount of energy of the focal spot (25 *μ*m) of the guided beam at the entrance of the plasma channel. The error bars represent the standard deviation over an average of shots.

The delayed guided beams are injected into the waveguides and the exit modes image relayed onto a CCD camera, as described in the previous section. Figure 4 shows the exit modes of the laser for several delays between the channel and guided pulses. The two top images show the intensity profiles of the guided beam at the exit and entrance (left and right) of the nozzle when there is no initial plasma (vacuum). The guided beam (Fig. 4, top right) is focused at the entrance of the gas jet with a spot size of 25 *μ*m corresponding to an intensity of 2 × 10^15^ W cm^−2^. The rest of the images in Fig. 4 show the guided beam intensity profile at the waveguide exit for several delays between the guided and the channel pulses. In this case we use a backing pressure of 20 bar (best guiding results). A polarizer cube allows imaging of only the guided beam on the CCD camera, because the guided and channel beams are cross polarized. Therefore, the images in Fig. 4 only show the guided light through the plasma waveguides. The guiding efficiency and intensity of the guided beam are determined using images such as those shown in Fig. 4. Figure 5 shows the amount of light transmitted for several delays between the channel and guided beams. The transmission is calculated by considering the encircled energy in a radius of 25 *μ*m at the exit of the waveguide, normalized to the energy contained in the vacuum spot size (25 *μ*m) of the guided beam at the entrance of the plasma waveguide.

The highest transmitted intensity is 0.74 × 10^15^ W cm^−2^ at 2 ns delay, with 35% guiding efficiency over more than 4 Rayleigh lengths (Rayleigh length is 1.8 mm for a 25 *μm* spot size) and the guided mode is quite stable from shot to shot with a standard deviation of 5.4% over 12 consecutive shots. The transmission at 2 ns delay is the highest because the matched spot size of the channel^13^ of 27 *μm* is very close to the guided beam spot size of 25 *μm*. At shorter delays the energy transmitted is lower and at longer delays the mode becomes larger (Fig. 4).

**Figure 6:**
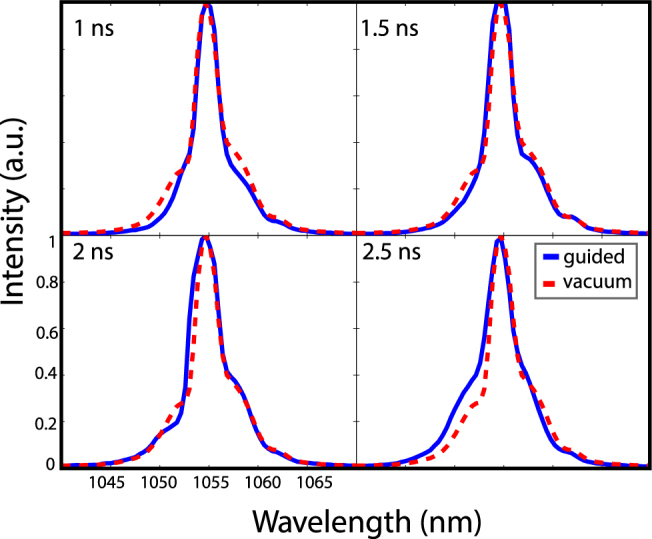
Comparison of spectra of the guided beam at the exit of the plasma waveguide (blue solid line) with the spectrum of the guided beam in vacuum (red dashed line) for several delays between the channel and guided beams.

As mentioned in the experimental setup the spectral content of the guided beam has been measured using a spectrometer. Figure 6 shows the input and output spectra of the guided beam for several delays. Apart from slight deviations there is no significant change in the spectral content of the guided beam. Although the guided beam intensity is enough to ionize the hydrogen gas the lack of an ionization-induced- blueshift indicates that the guided beam does not cause significant additional ionization of the waveguide.

The amount of transmitted light can be increased, but is being limited by two factors: the spot quality of the guided beam and the tapering of the plasma waveguide at the entrance and exit. With the current experimental setup we were not able to further improve the quality of the guided beam spot. However, if the spot quality is improved more energy can be coupled into the waveguide thus increasing the transmission. The second reason, tapering at the waveguide ends, results in inefficient coupling of the injected pulses. This is due to the gas density ramps at the edges of the gas jet^26^, where the reduced density results in a different radial expansion rate of the waveguide ends. In our case the gas jet has 1.5 mm density ramps at both ends^27^ leaving a uniform density region of 5 mm. A solution for this problem is to reduce the density ramps of the gas jet by using a gas cell. With a gas cell the density ramps are of the order of the cell entering diameter. In our case the channel beam has a diameter of 100 *μ*m, thus the density ramps would be decreased 15 times, from 1500 *μ*m to 100 *μ*m, increasing the guiding efficiency of the guided beam and the amount of transmitted light. Additionally the use of a gas cell is also a solution to increase the length of the plasma waveguide. Also it as been shown that the use of circular polarized light to generate a plasma waveguide is beneficial since it increases the initial temperature of the plasma^29^ when compared to linear polarization. This will generate deeper channels increasing the coupling and efficiency of the waveguide.

## Discussion

We have demonstrated that this novel scheme has the unique ability to guide a high-intensity laser pulse in a plasma waveguide created by the same laser system in a very simple and stable experimental setup. A hot plasma column is created by a femtosecond laser that heats the plasma to initial temperatures of tens of eVs^25^. After a few hundred picoseconds the plasma begins to expand and after 1 ns a clear shock structure is formed at the edges of the plasma, leaving a parabolic low density profile suitable to act as a waveguide for high-intensity laser beams. We have successfully guided ~10^15^ W cm^−2^ laser pulses in a 8 mm long hydrogen plasma waveguide. The highest transmission is achieved at a 2 ns delay, where the laser beam spot is matched to parabolic plasma channel. The guided mode was found to be very stable on a shot-to-shot basis with a 5.4% standard deviation over more that 12 consecutive shots and with a guiding efficiency of 35%. These novel guiding scheme has two major advantages. First because it is based on hydrogen gas, it is not possible to further ionize the gas making these waveguides very attractive for guiding very intense laser pulses, for laser wakefield accelerators. Second, because the matched spot size is two times smaller when compared to the common waveguides created by electric discharges, it is possible to use moderate power laser systems to achieve the necessary laser intensities to created LWFAs. Moreover, this scheme, as opposed to the current methods, uses one femtosecond laser system focused on a gas jet, simplifying the experimental apparatus and improving immunity to pointing stability errors.

## Methods

### Experimental Setup

**Figure 7:**
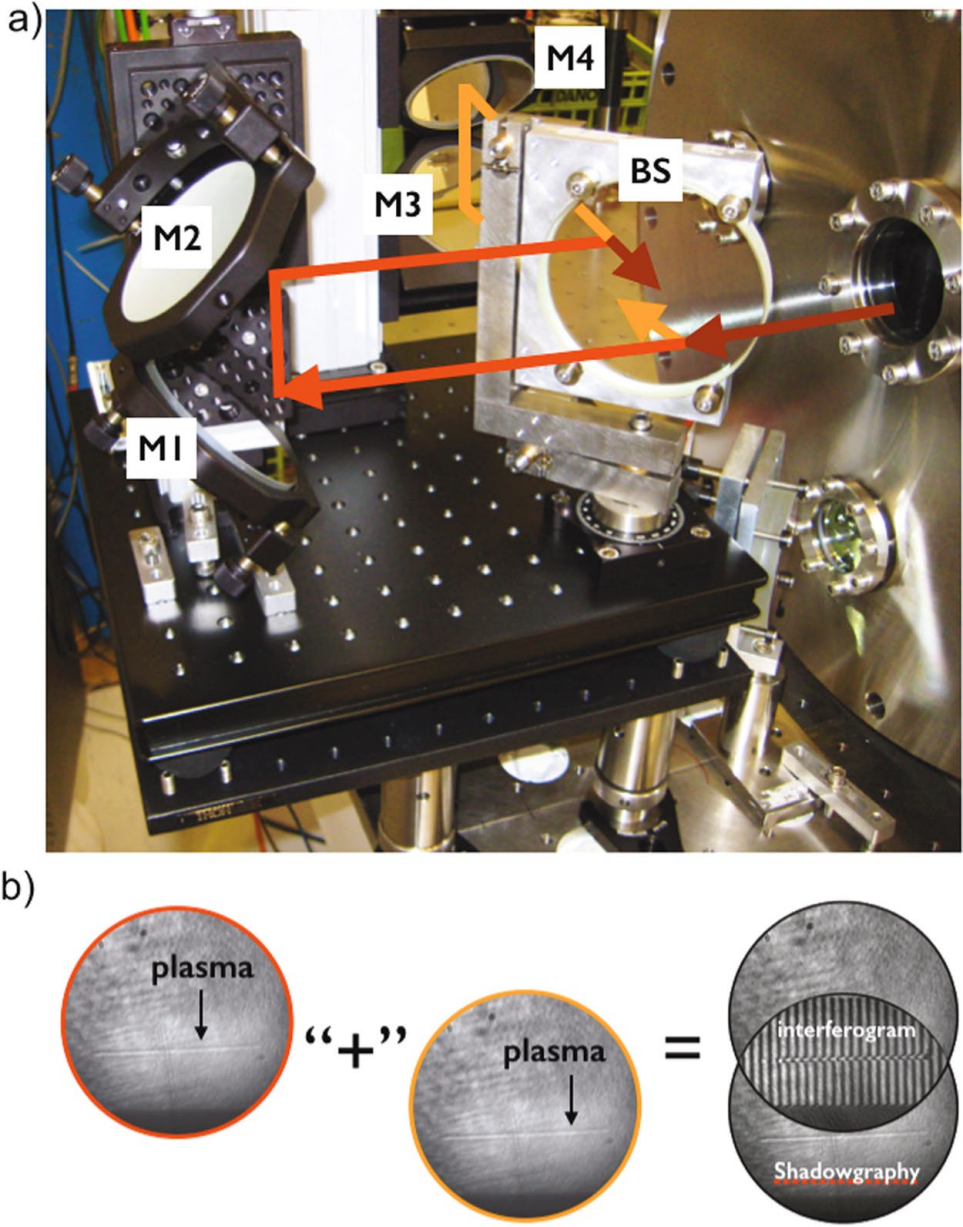
(**a**) Folded interferometer experimental setup. (**b**) Overlap of the two beams from the interferometer to generate an interferogram and a shadowgram.

A folded interferometer was used to obtain both shadowgraphy and interferometry for the same shot. A double breadboard with rubber vibration dumps was used to isolate the interferometer from external vibrations. Figure 7(a) shows a picture of the interferometer where the probe beam (dark red) comes from the chamber and is divided into two equal beams by a beam splitter (BS). One beam (red) goes into M1 and the other (orange) goes into M3 and will recombine back in the BS after the round trip to generate the interferometry pattern (Fig. 7(b)). Since the distance between M1 and M2 is smaller than the distance between M3 and M4 the beams will only partially overlap (Fig. 7(b)) generating an interferogram and shadowgraphy of the plasma. The plasma column shadowgraphs are imaged onto a digital CCD camera (14 bit) using a telescope with a magnification of 1:1.7, resulting in a resolution of 7.65 *μ*m/pixel. This magnification is used for imaging a 8 mm nozzle into the CCD camera. The interferograms are imaged onto a digital CCD camera (10 bit), with a magnification of 6:1 and a resolution of 0.75 *μ*m/pixel. An interferogram analysis code was used to determine the plasma density profiles for each probe beam delay. The code first, retrieves the phase shift from the interferogram, and then the refraction index from the phase shift. To obtain the phase shift we applied an automatic method based on two-dimensional FFTs^27^. The phase-shift resolution for the phase maps is 0.03 rad. The phase shift caused by the plasma is then obtained by subtracting the phase maps of a reference interferogram (without plasma) from the phase given by the interferogram with plasma. The electron density is calculated by inverting the 2D phase maps using an Abel inversion algorithm. A high resolution is required to resolve the plasma in the transverse direction to reduce the noise generated by the Abel inversion used to obtain the density profiles from the interferograms phase maps. The drawback of this high magnification is that we can only image a 1 mm portion of the total plasma column into the CCD, thus interferometry only images the central part of the nozzle.

The hydrogen gas jet is produced by an 8 mm Mach 6 Laval nozzle inside a 1 m^3^ vacuum chamber that was kept at a base pressure of 10^−2^ mbar by a pumping system. A fast solenoid valve opens long enough to produce a gas jet of constant flow, that has been determined to be optimal at 50 ms after valve opening. The backing pressure is varied from 10 to 35 bar and the neutral density, 1 mm from the tip of the nozzle, ranges from 1 × 10^18^ to 5 × 10^18^ cm^−3^. The Hagena parameter^31^ for this nozzles and gas is kept below 1000 so that no significant clusters are present in the gas jet flow. Hydrogen gas was chosen to generate the plasma waveguides since it is easy to fully ionize and prevents any extra ionization caused by the guided beam maximizing the transmission throughout the waveguide.
